# S-100*β* and Antioxidant Capacity in Cerebrospinal Fluid during and after Thoracic Endovascular Aortic Repair

**DOI:** 10.1155/2017/6875195

**Published:** 2017-06-27

**Authors:** Koichiro Nandate, Deepak Sharma, Hernando Olivar, Matthew Hallman, Ramesh Ramaiah, Aaron Joffe, Anthony Roche, Vijay Krishnamoorthy

**Affiliations:** Department of Anesthesiology and Pain Medicine, University of Washington, Seattle, WA, USA

## Abstract

**Background:**

Thoracic Endovascular Aortic Repair (TEVAR) has substantially decreased the mortality and major complications from aortic surgery. However, neurological complications such as spinal cord ischemia may still occur after TEVAR. S-100*β* is a biomarker of central nervous system injury, and oxidant injury plays an important role in neurological injury. In this pilot study, we examined the trends of S-100*β* and antioxidant capacity in the CSF during and after TEVAR.

**Methods:**

We recruited 10 patients who underwent elective TEVAR. CSF samples were collected through a lumbar catheter at the following time points: before the start of surgery (*T*0) and immediately (*T*1) and 24 (*T*2) and 48 hours (*T*3) after the deployment of the aortic stent. S-100*β* and CSF antioxidant capacity were analyzed with the use of commercially available kits.

**Results:**

We observed that the level of S-100*β* in all of the subjects at 24 hours after the deployment of the aortic stent (*T*2) increased. However, the levels of S-100*β* at *T*1 and *T*3 were comparable to the baseline value. The antioxidant capacity remained unchanged. No patient had a clinical neurologic complication.

**Conclusions:**

Our observations may indicate biochemical/subclinical central nervous system injury attributable to the deployment of the aortic stent.

## 1. Background

The development of postoperative paraplegia and stroke are major complications of open surgery (with use of either cardiopulmonary bypass or aortic cross-clamping) for repair of thoracic aortic aneurysms [1]. In recent years, Thoracic Endovascular Aortic Repair (TEVAR) and stenting of aortic aneurysms have largely replaced the open procedure. While the endovascular approach is associated with an overall reduction in perioperative morbidity, it has not successfully reduced the incidence of postoperative neurological complications, which has been reported at up to 12.5% [2–4].

While many clinical risk factors for neurologic complications (such as age, preoperative neurological status, stent length, and spinal cord perfusion pressure) have been identified [5–10], validated biomarkers of neurologic injury are lacking. Knowledge of biochemical changes and their relationship to neurologic injury during TEVAR may be useful for predicting neurological injury and instituting preventive measures [11]. S-100*β* has been shown to be a sensitive marker to detect spinal cord injury [12]. S-100*β* is a calcium binding protein found in high concentrations in glial and Schwann cells in the central nervous system, and it is released during acute injury to the spinal cord. Generated oxygen radicals and oxidant stress have also been appreciated to play a pivotal role in paraplegia secondary to spinal cord injury [13–15]. Direct evidence of oxidant-mediated tissue injury would be ideal, but it is unrealistic to have spinal cord tissue for analysis in this surgery; thus, an alternative method is to monitor antioxidant capacity. In this prospective, observational, pilot study we examined serial changes in S-100*β* (a biomarker of neurologic injury) and Trolox (an expression of antioxidant capacity) in the cerebrospinal fluid (CSF) during and after elective TEVAR surgery.

## 2. Methods

### 2.1. Ethics

After approval by the Institutional Review Board (IRB) and informed written consent, we recruited 10 patients who underwent elective TEVAR surgery.

### 2.2. Clinical Care

General anesthesia was induced with propofol and fentanyl, and anesthesia was maintained with a balanced anesthetic technique of sevoflurane with fentanyl and hydromorphone. A 22-guage catheter was inserted through the right radial artery to monitor blood pressure, and two large intravenous peripheral catheters were inserted. Also, after the induction of general anesthesia, under strict aseptic conditions, a drainage catheter (EDM Lumbar Catheter, Medtronic NJ, USA) was inserted into the subarachnoid space at the 3rd or 4th lumber space—this was used to monitor the CSF pressure and drain CSF during and after the surgery, if necessary.

In accordance with the institutional protocol, the mean arterial pressure was maintained within 10% of baseline for each patient before the deployment of the stent. Immediately after stent deployment, the mean arterial pressure was increased by approximately 20 mmHg. The drainage of CSF was initiated when the CSF pressure exceeded 10 mmHg. The CSF was drained at a rate up to 10 ml/hour until the pressure decreased below 10 mmHg. After the completion of surgery (surgical team confirmed the position of the stent in the descending aorta), the patients were emerged from general anesthesia. In the postanesthesia care unit and intensive care unit, the CSF perfusion pressure was maintained >80 mmHg by controlling mean arterial pressure and CSF pressure, using fluids and vasopressors/inotropes as appropriate. In the absence of neurological symptoms, drainage was stopped at 24 hours after surgery and the catheter was removed at 48 hours after surgery.

### 2.3. CSF Analysis

Three ml of CSF fluid was withdrawn from the lumbar drain before the start of surgery (*T*0), immediately after aortic stent deployment (*T*1), 24 hours after stent deployment (*T*2), and 48 hours after stent deployment (*T*3). After obtaining CSF, the samples were sent for immediate freezing at −80°C and were maintained at this temperature (following regular checks to ensure consistent and steady freezing) until they were analyzed. S-100*β* was measured by enzyme-linked immunosorbent assay (ELISA) with use of a commercially available kit (DRG, Germany). Antioxidant Capacity was analyzed by colorimetric assay with use of commercially available kit (Cayman Chemical, Ann Arbor, USA). This method is based on the interaction of the phenothiazine compound 2,2′-azino-di[3-ethylbenthiazoline sulphonate] (ABTS) with ferryl myoglobin, produced through the reaction of the ABTS^•+^ radical, which can be monitored by reading absorbance at 750 nm or 405 nm. In the presence of antioxidants, the absorbance of this radical cation is quenched to an extent directly related to the concentration of antioxidants present in CSF sample. The extent to which the antioxidants in the sample solution inhibited the reaction was calibrated with use of a standard antioxidant solution.

### 2.4. Statistical Analysis

Data are expressed as proportions (percent) and median (interquartile range). We used paired *t*-tests (with a Bonferroni correction due to multiple testing) to test for a statistically significant difference in S-100B and Trolox levels postoperatively, at 24 hours, and 48 hours, compared to preoperative levels. We then used an analysis of variance (ANOVA) to test for an overall difference in S-100B levels between time points. A statistical result of *p* < 0.017 (0.05/3 per the Bonferroni correction for 3 total tests) was considered significant for the paired *t*-test analyses, while a level *p* < 0.05 was considered significant for the ANOVA analysis. All analyses were conducted using STATA 13.0 (College Station, TX).

## 3. Results

Our cohort consisted of ten patients with a median age of 67 (63–74) years; among this cohort, 6 (60%) were male. The demographic data of the patients is summarized in Table 1.

**Figure 1:**
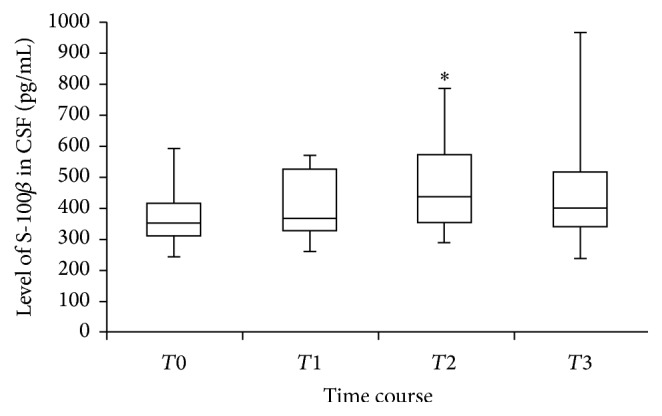
S-100*β* levels over time after the deployment of the stent. Median, 25th and 75th percentiles, and ranges (vertical bars) of S-100*β* level of cerebrospinal fluids. *T*0 = before the surgery start (baseline), *T*1 = immediately after the deployment of the aortic stent, *T*2 and *T*3 = 24 and 48 hours after the deployment of the aortic stent (^*∗*^*p* < 0.05 compared to *T*0).

The CSF level of S-100*β* was significantly higher at 24 hours after the deployment of the aortic stent (*T*2), compared to preoperative levels (*p* = 0.007). S-100*β* levels were increased at 24 hours in all 10 patients. However, the S-100*β* levels postoperatively (*p* = 0.04) and at 48 hours (*p* = 0.10) after the deployment of the stent did not differ significantly (based on our predefined statistical threshold) compared to baseline, although the postoperative level trended toward significance (Figure 1). Using an ANOVA analysis, there was a significant trend for differences in S-100*β* biomarkers levels over the entire time period (*p* = 0.023).

**Figure 2:**
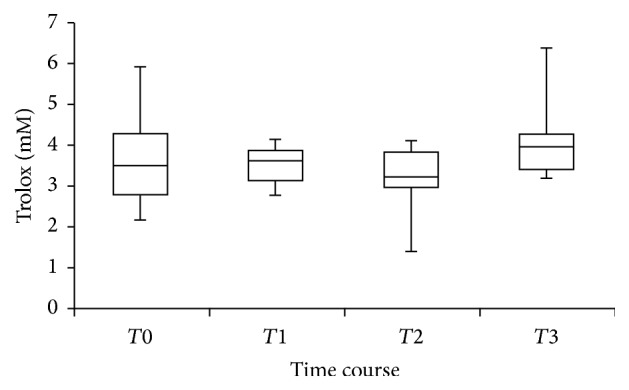
Trolox (vitamin E analogue) levels over time after the deployment of the stent. Median, 25th and 75th percentiles, and ranges (vertical bars) of Trolox levels cerebrospinal fluids. *T*0 = before the surgery start (baseline), *T*1 = immediately after the deployment of the aortic stent, *T*2 and *T*3 = 24 and 48 hours after the deployment of the aortic stent.

Antioxidant capacity in the CSF (expressed as Trolox) did not change significantly after stent deployment compared to baseline (*p* = 0.79), at 24 hours (*p* = 0.40) or at 48 hours (*p* = 0.20) (Figure 2).

No patients had neurologic complications after surgery, and the postoperative neurological examinations remained unchanged compared to the preoperative status.

## 4. Discussion

In summary, our results show that, in patients undergoing TEVAR, the S-100*β* levels in the CSF increased significantly at 24 hours after stent deployment but returned to baseline after 48 hours. In addition, the antioxidant Trolox levels did not change during the 48 hours after TEVAR. This data, coupled with clinical observations in the postoperative period, indicate that most patients experience mild central nervous ischemia following stent deployment, but it generally resolves and usually does not lead to clinically relevant neurologic insults. By following the dynamics of S-100*β* and antioxidant capacity, our study adds to the knowledge of when the spinal cord is exposed to ischemia after TEVAR. This may help guide protocols for CSF monitoring and drainage during and after TEVAR.

S-100*β* is a calcium binding protein which is located selectively in glial and Schwann cells and is therefore unique to the central nervous system. S-100*β* in CSF has previously been identified as a specific and reliable biomarker for central nervous system injury [12]. Brunnekreef et al. have explored S-100*β* in the CSF of the patients whom they considered higher risk for postoperative neurological dysfunction; however, S-100*β* in CSF did not significantly increase throughout their sampling points [16]. This could be due to differences in age, gender, stent length, and clinical management in their patient population.

Our result indicates that S-100*β* tends to increase over 48 hours after stent deployment but only reached statistical significance at 24 hours. However, the clinical significance of this increase of S-100*β* is unclear because even the maximum level of CSF S-100*β* in this study was below 1.0 microgram/L, the previously identified upper limit of CSF S-100*β* levels in subjects without any neurological symptoms [17]. Additionally, none of the patients enrolled in this study had clinically apparent neurological complications, and endogenous antioxidant capacity was unchanged throughout the study period. When combined with the lack of clinical adverse events, this may indicate that no significant oxidative stress was generated. However, it should also be noted that a recent study demonstrated a high incidence of new diffusion restriction foci on cerebral DW-MRI in a pattern suggestive of embolization despite a lack of symptoms [18]. As we did not perform neuroimaging as part of this study, we are unable to comment on the possible association of the transient increase in S-100*β* with MRI findings. Importantly, CSF was drained in all patients and hemodynamics were managed to maintain the spinal perfusion pressure >80 mmHg and this might have prevented the development of persistent spinal ischemia/neurological deficits.

The central nervous system is very vulnerable to ischemia, due to its nature of high metabolic requirement for oxygen. CNS ischemia initiates a complicated cascade of events that culminate in oxidant generation. This response is normally balanced by an increase in the production of endogenous antioxidants including enzymes such as superoxide dismutase and glutathione peroxidase. Therefore, antioxidant production has been considered a logical surrogate for ischemic injury [15–17]. While our results suggest the possibility of mild spinal cord ischemia (based on elevated S-100-B levels), this did not result in increasing antioxidant capacity in the spinal cord (based on unchanged Trolox levels).

There are several limitations to our study. First, the lack of neuroimaging may have precluded the ability to study the association of S-100*β* with MRI findings of ischemia, rather than relying on the clinical exam alone. Second, our small sample size makes it difficult to have statistical estimates of a high precision. Lastly, variations in patient demographics, anesthetic techniques, and surgical care may have contributed to the results that we observed.

## 5. Conclusion

This pilot study demonstrates that CSF levels of S-100*β* increased significantly 24 hours after stent deployment for thoracic aorta repair, but it did not reach a clinically significant value. Endogenous antioxidant capacity as measured by Trolox levels was unchanged throughout the study period. Therefore, our study adds to the mechanistic data of the development of SCI after TEVAR, but further studies are needed to elucidate the clinical relevance of our findings. Future research should employ larger samples sizes with more frequent sampling points to evaluate clinical outcomes and study the association of CSF biomarkers with neuroimaging abnormalities.

## Figures and Tables

**Table 1:** Demographic and clinical characteristics (*n* = 10).

Characteristic	Data
Gender (M : F)	6 : 4
Age (years)	67 (63–74)
Height (cm)	176.4 (167.9–187.3)
Weight (kg)	78.5 (68.9–93.2)
Anesthesia duration (min)	205 (183–220)
Surgery duration (min)	86 (76–115)
Blood loss (mL)	50.0 (20.0–53.8)
Intraoperative urine output (mL)	475 (325–825)
